# Arthroscopic All-Inside Posterior Cruciate Ligament Avulsion Fracture Suture Fixation With Double-Tunnel Pullout and High-Strength Suture Tape Augmentation Using Trans-septal Approach

**DOI:** 10.1016/j.eats.2024.103091

**Published:** 2024-08-03

**Authors:** Marcello Capella, Davide D’Antonio, Luca Drocco, Luca Barberis, Daniele Vezza, Fortunato Giustra, Alessandro Massè

**Affiliations:** a School of Medicine, University of Turin, CTO Hospital, Turin, Italy; b Humanitas Gradenigo Hospital, Turin, Italy; c San Giovanni Bosco Hospital, Turin, Italy

## Abstract

This technical note aims to provide a detailed description of our arthroscopic technique for suture fixation of posterior cruciate ligament (PCL) tibial avulsion fractures. Various surgical approaches have been described, including both open and arthroscopic techniques. The arthroscopic approach can be less disruptive and more accurate in visualizing anatomic landmarks. It also may ensure good and reliable fracture reduction and fixation. This article describes a technique for arthroscopic all-inside fixation of PCL avulsion fractures using a double-tunnel pullout method. The procedure involves trans-septal visualization and whipstitching suturing of the PCL with 1.5-mm high-strength, nonresorbable transverse ribbon suture (LabralTape; Arthrex, Naples, FL) and a crosstie-like suture with a high-strength, nonresorbable suture tape (FiberTape; Arthrex) embracing the PCL from anterior to posterior. Finally, the avulsion fracture is secured by tensioning a high-strength suture tape from the femur to the tibia along the PCL structure as an InternalBrace ligament augmentation (Arthrex). This technique allows for anatomic reduction and stable fixation of the displaced fracture through optimal trans-septal visualization and the PCL whipstitching technique, further secured by the InternalBrace ligament augmentation, enabling early and intensive rehabilitation.

Posterior cruciate ligament (PCL) avulsion fractures require prompt management to prevent nonunion of the fragment, chronic posterior instability, PCL footprint bone loss complicating secondary reconstruction, and post-traumatic arthritis.^1^^,^^2^ The preferred method of treating these conditions is generally considered open reduction and internal fixation.^3^^,^^4^ However, arthroscopic approaches have also been described, providing less invasive procedures, better visualization of anatomic landmarks, and treatment of associated intra-articular lesions.^5^

This technical note describes an updated arthroscopic PCL fracture fixation technique. This technique involves developing a trans-septal (TS) approach. The PCL substance is whipstitched with a high-strength ribbon suture, and a high-strength suture tape is used to embrace the PCL from anterior to posterior and then configured in a crosstie fashion. Additionally, an InternalBrace ligament augmentation (IBLA) (Arthrex, Naples, FL) is placed along the PCL course. The primary strength of this technique is that it provides anatomic and stable fixation of the PCL through combined high-strength sutures, enhanced by TS visualization. Moreover, no posterior hardware is required, and the IBLA system allows for early functional recovery of the knee.

## Surgical Technique

### Preoperative Evaluation

**Fig 1:**
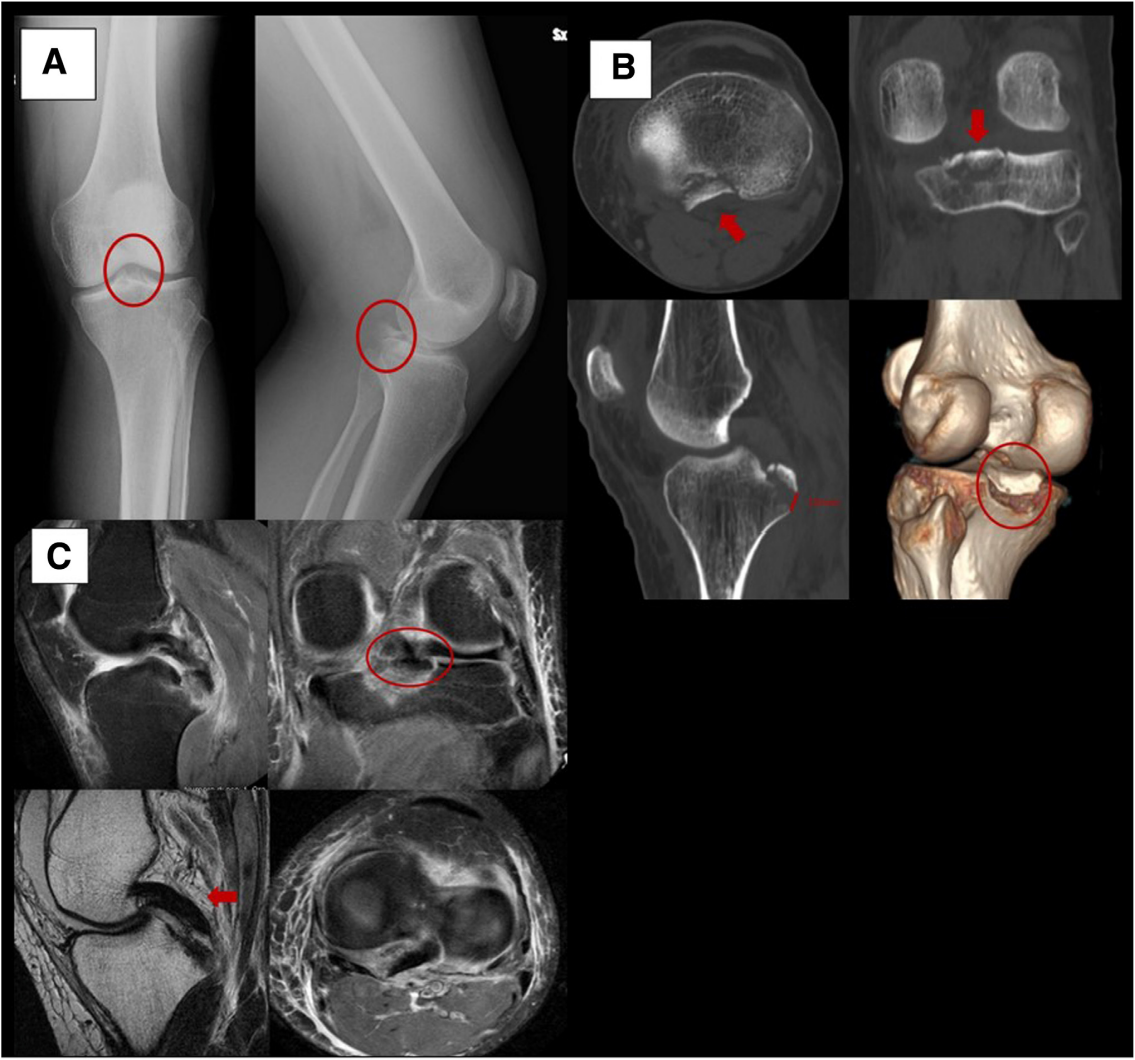
Preoperative imaging of left knee. (A) Anteroposterior and lateral radiographs showing isolated posterior cruciate ligament (PCL) avulsion fracture (ovals). (B) Axial, coronal, sagittal, and 3-dimensional computed tomography scans of same PCL avulsion fracture. Computed tomography reveals a multifragmentary fracture (arrows and red circle) with a major fragment and displacement of 10 mm, which requires surgery. (C) Sagittal, coronal, and axial magnetic resonance imaging (MRI) of same isolated PCL avulsion fracture. MRI reveals the continuity of the PCL substance signal (arrow) and edema at the fracture level (oval). MRI enables the identification of any associated meniscal or ligamentous lesions, as well as intra-articular free bodies.

After clinical evaluation, a 2-plane radiograph and a knee computed tomography scan are required to assess PCL footprint displacement, fragment characteristics, and associated fractures. Knee magnetic resonance imaging is mandatory to identify any associated soft-tissue injuries (Fig 1).

### Patient Positioning

**Fig 2:**
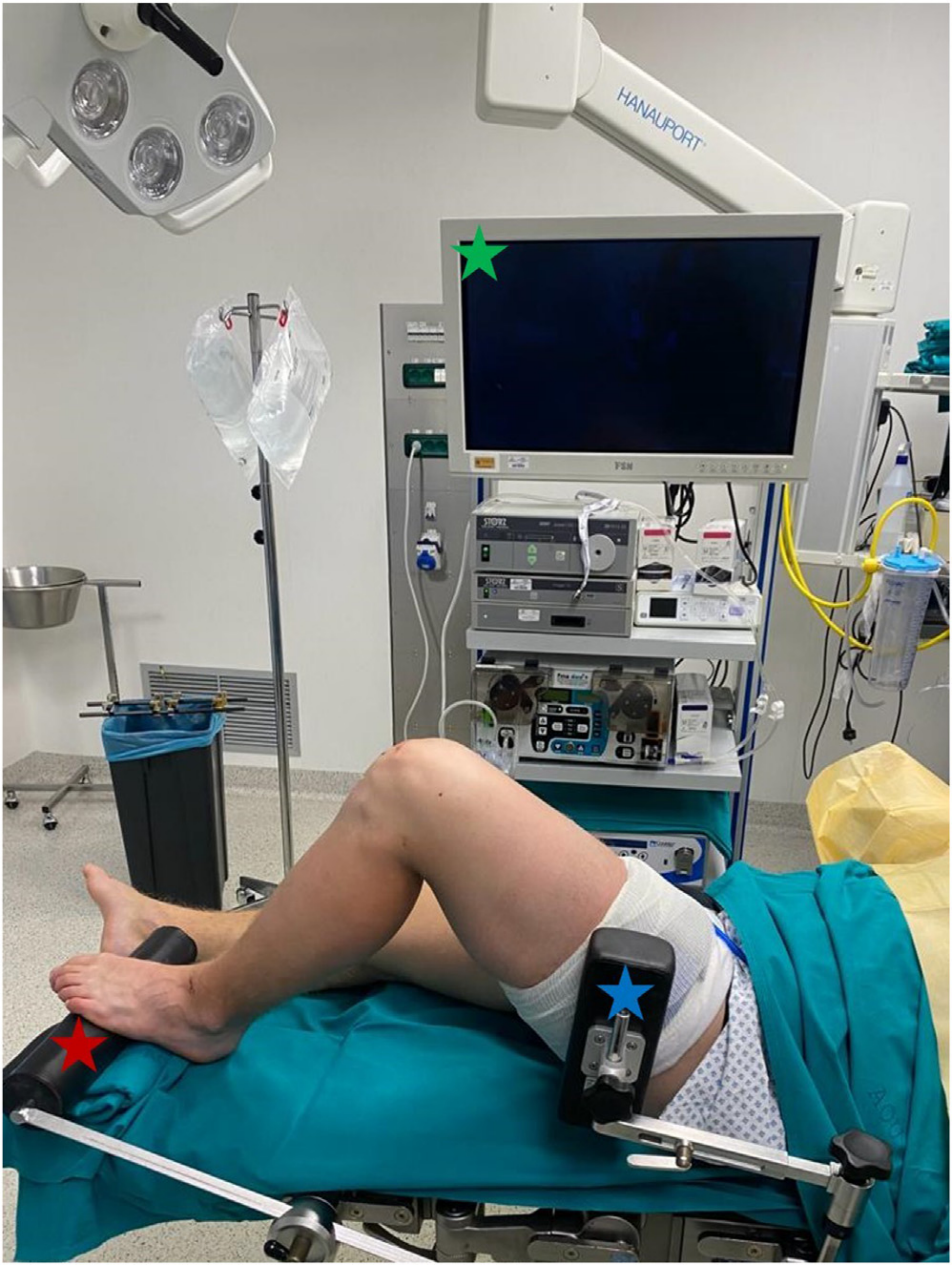
Operating room setup and patient positioning. The patient is positioned supine on the operating table with a transverse foot support (red star) and a lateral support (blue star) maintaining the knee at 90° of flexion. A tourniquet is placed around the thigh; the arthroscopic column (green star) is positioned on the contralateral side to the injured knee.

The procedure is shown in Video 1. The patient is positioned in the supine position on the operating table. The knee is flexed to 90° during the procedure through a transverse leg holder. Additionally, a lateral limb support and a contralateral pelvic support are applied to allow knee stress maneuvers during the management of associated lesions. At the beginning of the procedure, a tourniquet is placed around the thigh and inflated (Fig 2).

### Diagnostic Arthroscopy and TS Visualization Development

Standard infrapatellar lateral and infrapatellar medial (IPM) portals are developed (Table 1). A diagnostic arthroscopy is performed using a 30° camera to address associated lesions.

**Table 1:** Operating Room Setup, Patient Positioning, and Surgical Steps for Arthroscopic All-Inside PCL Avulsion Fracture Suture Fixation With Double-Tunnel Pullout and InternalBrace Augmentation Using Trans-septal Approach

Operating room setup and patient positioning:
Place the arthroscopy tower on the opposite side of the affected knee.
Position the patient supine on the radiolucent table with a transverse foot support and a lateral support to maintain the knee at a 90° angle and allow for stress maneuvers during the management of associated lesions.
Surgical steps:
Perform a standard diagnostic arthroscopy, and manage any associated lesions.
Create PM and PL portals via trans-notch visualization.
Pass the septum under direct trans-notch visualization from lateral to medial with the knee at 90° of flexion.
Develop the trans-septal portal by dissecting the septum until exposing the avulsed PCL footprint.
Use a DogBone cannulated reamer to drill two 3-mm transtibial tunnels at the medial and lateral borders of the PCL footprint with the aid of a PCL guide handle. Place 2 suture shuttles in the tunnels.
Pass the PCL substance with a 45° angled suture hook. Position an intrasubstance LabralTape with the aid of a suture shuttle, and retrieve the tips from the PL and PM portals.
Introduce the tip of the FiberTape into the posterior compartment of the knee, passing in an inter-cruciate manner.
Pass the second tip of the FiberTape in the posterior compartment of the knee, passing on the medial side of the PCL, thus embracing it.
Retrieve the tips of the FiberTape in a crosstie fashion from the PL and PM portals.
Use a 4-mm cannulated reamer to drill a tunnel centered on the femoral footprint of the PCL with the aid of a femoral PCL guide handle.
Load a FiberTape onto a TightRope RT. Retrieve the TightRope RT in the femoral tunnel, and flip the RT button on the medial femoral cortex.
Retrive the FiberTape InternalBrace into the PL portal with a Kingfisher clamp from the anterior compartment passing through the intercondilar notch.
Note that, at this point, the InternalBrace sutures, the lateral LabralTape tip, and one crosstie FiberTape tip are in the PL portal while the medial LabralTape tip and the second crosstie FiberTape tip are in the PM portal.
Retrieve all the sutures through the tunnels on the anterior tibial cortex using the previously positioned suture shuttles.
Keeping the knee at 90° of flexion, use an 11-mm ABS button to secure the sutures on the anterior tibial cortex.
First, tension and knot the InternalBrace system; perform cycling of the knee; and tension the InternalBrace system on the femoral side. Then, tension and knot the trans-cruciate LabralTape with a sliding knot, reducing the PCL into its footprint. Finally, tension and knot the crosstie FiberTape with a sliding knot, compressing the avulsed fragment.

PCL, posterior cruciate ligament; PL, posterolateral; PM, posteromedial.

The posteromedial (PM) and posterolateral (PL) portals are created using trans-notch visualization. An arthroscopic trocar or 4.2-mm shaver is introduced through the PL portal and advanced toward the posterior septum on the PL side, under trans-notch visualization. The septum is penetrated under direct visualization. To avoid posterior vascular bundle injury, TS portal development should be carried out with the knee at 90° to 100° of flexion and passing the septum in a lateral-to-medial direction^6^^,^^7^ (Table 2).

**Table 2:** Pearls and Pitfalls

Preoperative planning:
Management of injuries involving PCL avulsion fractures, concomitant diaphyseal femoral fractures, tibial plateau fractures, meniscal lesions, or multiligament knee injuries may be performed. It is crucial to assess the patient carefully and provide the correct indications and treatment.
Posterior portal development:
During posteromedial and posterolateral portal development, it is important to create a portal that is grossly parallel to the posterior aspect of the tibia and slightly anteriorly convergent. This will provide optimal visualization of the PCL footprint.
Trans-septal visualization development:
Care should be taken to pass the posterior septum remaining between the PCL fibers (anterior) and the capsule (posterior) to avoid incorrectly creating space in the posterior compartment. It is important to keep the knee at 90° of flexion and pass the septum from lateral to medial. This maneuver protects the posterior vascular bundle from accidental injury.
It is recommended to avoid proximal debridement of the septum because it is unnecessary and may result in potential injury to the middle geniculate artery.
During development of trans-septal visualization, the shaver should be used as a blunt dissector to displace the capsule posteriorly and identify the remaining fibers to be removed.
Tunnel preparation:
Dissection of the septum should be carried out distally to the footprint to ensure the correct positioning of the tunnels. The tunnels should diverge below the PCL attachment while converging on the anterior tibial cortex with a 5-mm bone bridge to allow for button fixation.
PCL whipstitching:
To prevent entanglement of the ACL and meniscofemoral ligaments in the suture that embraces the PCL, it is recommended to direct the KingFisher clamp adjacent to the tibial surface while passing through the notch.
Suture knotting:
For isolated PCL avulsion fractures, the surgeon should apply as much tension as possible on the sutures when performing closure with the knee at 90° of flexion. For multiligament knee injuries, the tension should be calibrated based on arthroscopic femorotibial congruence and anterior tibial tuberosity step-off.
Bleeding control:
At the end of the procedure, the surgeon should release the femoral tourniquet and visualize the posterior compartment of the knee. Minor bleeding may occur, particularly after injury to the middle geniculate artery. Any bleeding should be managed with the assistance of a radiofrequency device.

ACL, anterior cruciate ligament; PCL, posterior cruciate ligament.

**Fig 3:**
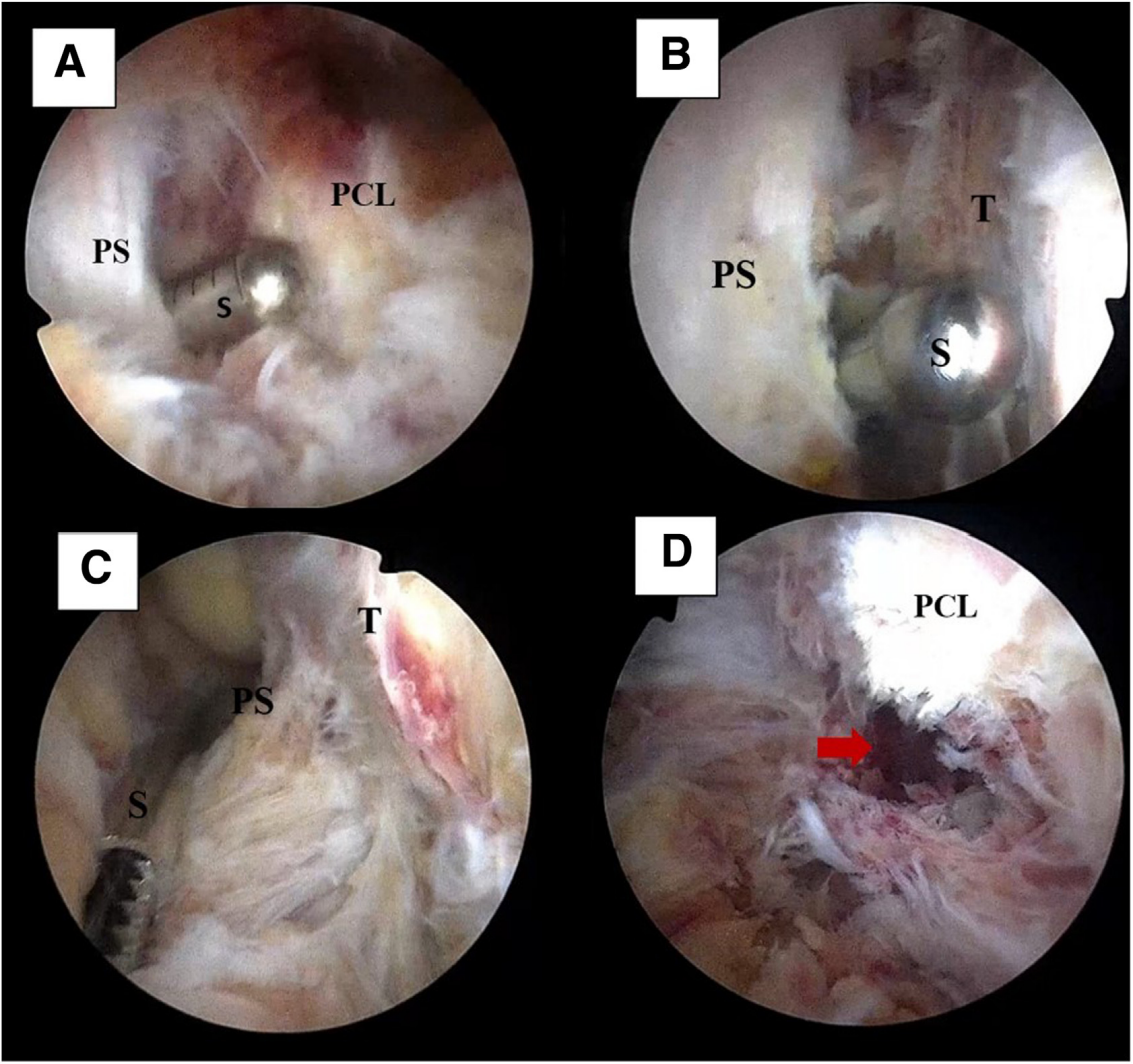
Intraoperative arthroscopic images of posterior compartment of left knee: trans-septal visualization development. Visualization is obtained from the posteromedial portal while a shaver is introduced through the posterolateral portal. (A) Intraoperative image of proximal posterior septum (PS) dissection. The shaver (S) is directed anteriorly toward the posterior border of the tibia. Care should be taken not to damage the posterior cruciate ligament (PCL) during the procedure. (B) Dissection should be carried out distally, with the shaver (S) moving down the posterior margin of the tibia (T). Care must be taken to avoid turning the shaver posteriorly to prevent injury to the posterior vascular bundle. (C) The shaver (S) is used as a blunt dissector to detach the PS fibers from the tibia (T) and to identify the remaining fibers to be removed. (D) The displaced PCL avulsion fracture (arrow) can be visualized and prepared for reduction.

The camera is positioned in the PM portal while the 4.2-mm shaver is used to dissect the posterior septum from the PL portal. The knee is kept flexed to 90°, and the shaver is directed toward the posterior border of the tibia. Space is created in the posterior compartment, developing TS visualization, and extended distally along the posterior edge of the tibia to visualize the avulsed PCL remnant (Fig 3).

### Tunnel Positioning and PCL Avulsion Whipstitching

A PCL guide handle (Arthrex) is inserted through the IPM portal and introduced in the posterior compartment passing through the intercondilar notch, lateral to the PCL remnant. The guide is positioned on the distal medial border of the PCL attachment site. With visualization through the PL portal, a 3-mm cannulated reamer (DogBone; Arthrex) is used to drill a transtibial tunnel from anterior to posterior, with the extra-articular part of the guide handle positioned on the anteromedial cortex of the tibia. To avoid posterior vascular bundle injury, it is important to protect the tip of the reamer with the PCL guide. The PCL guide is repositioned between the anterior cruciate ligament and the lateral femoral condyle. A second transtibial tunnel is similarly created at the distal lateral border of the PCL attachment. Two suture shuttles are positioned in the tunnels using 2 Chia nitinol loop passers (Zimmer Biomet, Warsaw, IN). A 45° angled suture hook (DePuy Mitek, Raynham, MA) loaded with a shuttle suture is inserted through the PL portal and passed through the PCL fibers from lateral to medial. The shuttle suture is then replaced with a 1.5-mm high-strength ribbon suture (LabralTape; Arthrex).

**Fig 4:**
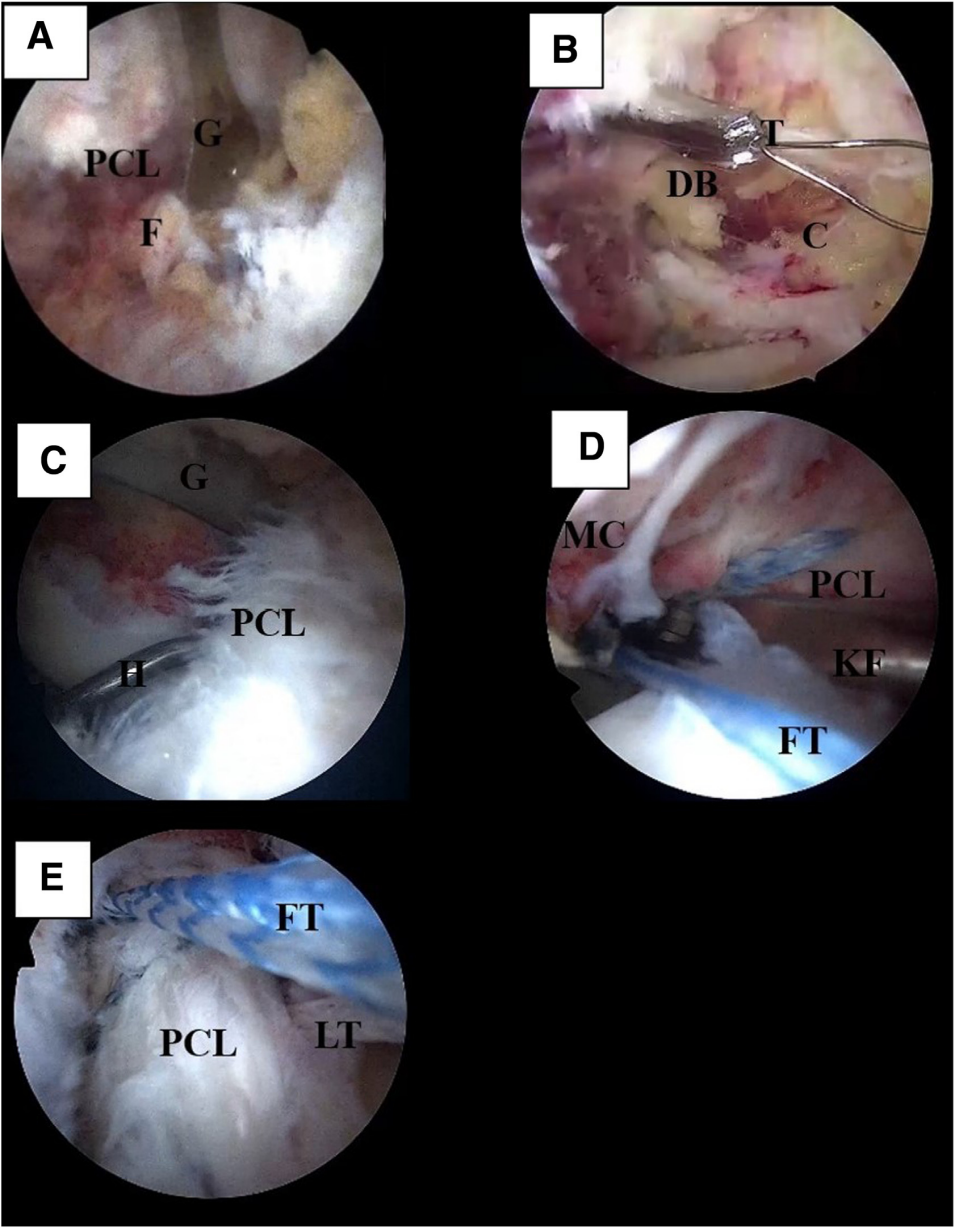
Tunnel positioning and posterior cruciate ligament (PCL) whipstitching in left knee: intraoperative arthroscopic images with visualization from posterolateral (PL) portal. (A) The PCL guide (G) is inserted through the infrapatellar medial portal, passed laterally to the PCL, and positioned at the medial lower edge of the PCL footprint (F). (B) A tunnel is drilled with a 3-mm cannulated reamer (DogBone [DB]), and a Chia nitinol loop passer (C) is passed from the cannulated reamer, retrieved through the posteromedial (PM) portal, and replaced with a shuttle suture. The same procedure is performed for lateral tunnel development, with the Chia wire being retrieved from the PL portal and then replaced by the shuttle suture. (C) A 45° angled suture hook (H) loaded with a shuttle suture is inserted through the PL portal and passed through the PCL fibers from lateral to medial. The PCL guide (G) is used as a spreader to optimize visualization of anatomic structures. The shuttle suture is replaced with a 1.5-mm high-strength ribbon suture (LabralTape [LT]), configuring the trans-cruciate suture. (D) With visualization from the infrapatellar lateral portal, a high-strength, nonresorbable suture tape (FiberTape [FT]) embraces the PCL remnant from anterior to posterior. The suture is introduced through the notch in the posterior compartment with a KingFisher clamp (KF), passing on the medial side of the PCL remnant, after having passed the first tip of the suture in an inter-cruciate manner on the lateral side of PCL remnant. The tips of the suture tape are retrieved in a crosstie configuration through the PL and PM portals. (E) Visualization of the final whipstitching configuration from the PM portal shows the PCL being embraced from anterior to posterior by the suture tape (FT) in a crosstie configuration and the trans-cruciate ribbon suture (LT). (MC, medial condyle; T, tibia.)

The tips of the ribbon suture are temporarily retrieved from the PL and PM portals, respectively. A high-strength, nonresorbable suture tape (FiberTape; Arthrex) is introduced from the IPM portal with a KingFisher clamp (Arthrex) and passed in an inter-cruciate manner on the lateral side of the PCL remnant. The second tip of the suture tape is then retrieved through the infrapatellar lateral portal and passed through the notch in the posterior compartment on the medial side of the PCL remnant, embracing the ligament from anterior to posterior. The suture tape tips are retrieved from the PM and PL portals in a crosstie configuration on the PCL (Fig 4).

### Establishment of IBLA

**Fig 5:**
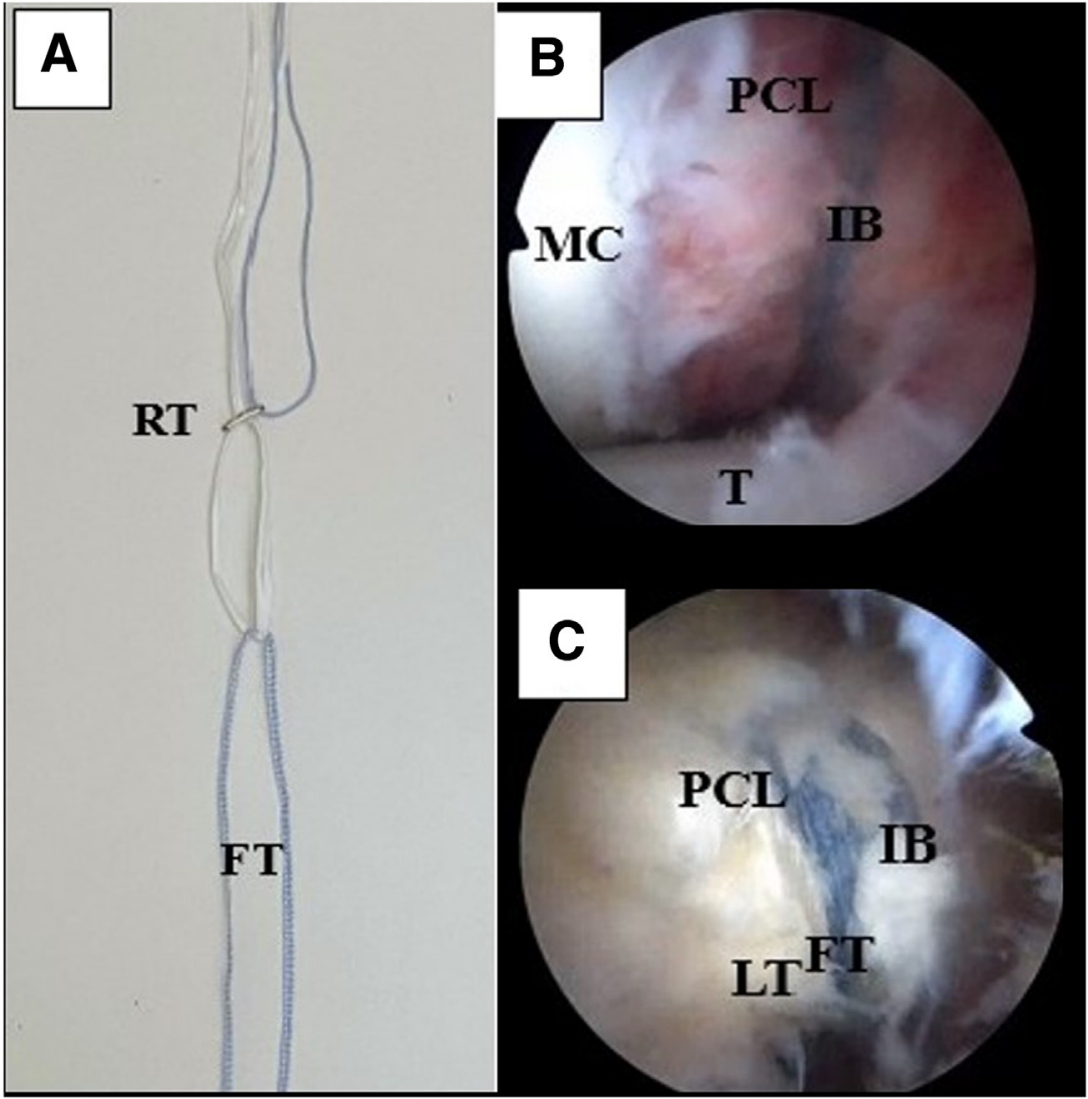
InternalBrace (IB) ligament augmentation system preparation in left knee. (A) Configuration of IB ligament augmentation system with high-strength, nonresorbable suture (FiberTape [FT]) straddling loop-adjustable cortical fixation device (TightRope RT [RT]). (B) Visualization from infrapatellar lateral portal. After the TightRope RT is secured on the medial femoral cortex, the InternalBrace (IB), straddling the adjustable loop, is transported in the posterior compartment of the knee, passing between the medial femoral condyle (MC) and posterior cruciate ligament (PCL). (C) The final suture fixation configuration is visible from the PL portal after passing the sutures through the tibial tunnels. The trans-cruciate suture ribbon secures the PCL into its footprint. The crosstie suture tape (FiberTape [FT]) embraces the PCL and compresses the avulsed fragment. Finally, the IB ligament augmentation protects the fixation. (LT, LabralTape; MC, medial condyle; T, tibia.)

A femoral out-in tunnel is drilled using a femoral PCL guide (Arthrex) and a 4-mm cannulated reamer. The tunnel reference point is the femoral PCL footprint. A high-strength, nonresorbable suture tape (FiberTape) is loaded onto a loop-adjustable cortical fixation system (TightRope RT; Arthrex). The TightRope RT is retrieved through the tunnel and the button is flipped on the femoral cortex under direct arthroscopic visualization. The free ends of the suture tape are passed into the posterior compartment through the notch and retrieved from the PL portal using a suture shuttle (Fig 5).

### Pullout and Fixation

**Fig 6:**
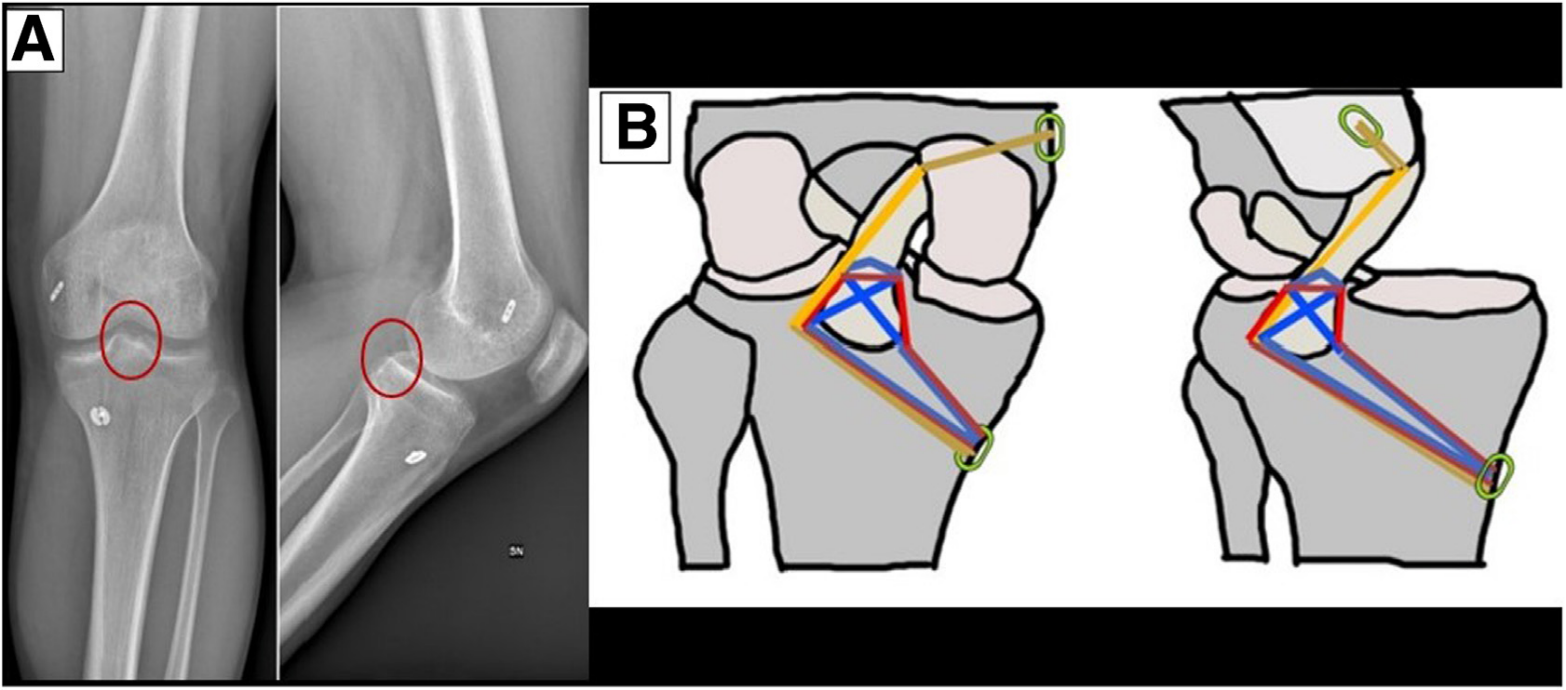
Left knee. Postoperative radiograph and summary of suture fixation technique. (A) The postoperative radiograph shows successful reduction and fixation of the avulsion fracture of the posterior cruciate ligament (PCL) using our procedure. The RT button (Arthrex) can be seen fixed to the medial femoral cortex, whereas the ABS button sits on the medial tibial cortex. Both the anteroposterior and lateral projections show excellent fracture reduction (ovals). (B) Summary of arthroscopic all-inside PCL avulsion fracture suture fixation with double-tunnel pullout and InternalBrace augmentation. The red suture represents the 1.5-mm high-strength ribbon suture (LabralTape), configuring the trans-cruciate suture; the blue suture represents the high-strength, nonresorbable suture tape (FiberTape) that embraces the PCL remnant from anterior to posterior and is configured in a crosstie fashion. The gold suture represents the InternalBrace ligament augmentation (IBLA) system. The IBLA is secured with a loop-adjustable cortical fixation device (TightRope RT) on the femoral side (green ovals) and is retrieved through the lateral tibial tunnel with the PCL sutures in the posterolateral (PL) portal. The tips of the sutures and the IBLA are secured onto an ABS button on the tibial side (green ovals).

The PCL sutures and IBLA tails are retrieved from the PL and PM portals, respectively, through the lateral and medial tunnels to the anterior tibial cortex using 2 previously positioned suture shuttles. An 11-mm cortical suspension button (ABS button; Arthrex) is used to secure the PCL sutures and IBLA on the anterior tibial cortex. With the knee at 90°, the IBLA is first tensioned and secured on the button and the TightRope RT is further tensioned on the femoral side. Next, the trans-cruciate suture ribbon is knotted with a sliding knot to secure the PCL into its footprint. Finally, the crosstie suture tape is knotted with a sliding knot to compress the avulsed PCL fragment (Fig 6).

### Postoperative Management

The rehabilitation program involves early mobilization from the first postoperative day. Full range of motion and muscular strengthening exercises of the quadriceps are performed. The main goal is the achievement of full extension within the first week after surgery. Toe-touch weight bearing with a full-extension knee brace is allowed. The extension knee brace is only required during ambulation. After 6 weeks of rehabilitation and a radiographic assessment, full weight bearing is allowed.

## Discussion

Arthroscopic procedures are gaining popularity in the management of PCL avulsion fractures. Several studies have shown that the results of the arthroscopic approach are similar to those of the open approach.^8^^,^^9^ The arthroscopic approach offers optimal anatomic landmark visualization, allowing for effective fracture reduction and precise tibial tunnel positioning, combined with the management of associated intra-articular lesions with a less invasive procedure. The previously described arthroscopic PCL avulsion fixation procedure combines different techniques to enhance both visualization and fixation of the PCL, ensuring immediate rehabilitation. Some authors have stated that achieving reliable visualization of fragment reduction is difficult through only the PM portal, even with a 70° arthroscopic optic.^1^ Conversely, TS visualization, through posterior septum debridement, provides a wide working space that aids visualization and reduction of the fragment, even with altered anatomy due to fracture-dislocation.^7^ There is no consensus on the optimal fixation method for avulsed PCL fragments.^1^^,^^2^ The use of screws requires an intact fragment of appropriate size. Conversely, sutured wire fixation can be performed regardless of fracture morphology and may reduce the need for subsequent removal of the implant. This method may also provide a higher fixation effect on the avulsed PCL fragment owing to the direct grasping on the ligament.^10^, ^11^, ^12^ This article presents a technique that combines a high-strength ribbon suture threaded through the PCL fibers with a high-strength suture tape straddling the PCL and set in the tibial tunnels in a crosstie configuration. The ribbon and tape sutures enable traction over the entire PCL substance and could improve pressure distribution on the avulsed fragment. The ribbon reduces the fragment into its footprint while the straddling crosstie tape compresses the fracture, resulting in high-strength fixation of the fracture.

The IBLA system acts as a tissue bridge that protects the fixation when uncontrolled stresses, such as hyperextension or hyperflexion of the knee, are applied to it.^13^^,^^14^ Thanks to the IBLA system, patients can perform a full range of motion and quadriceps activation without a PCL brace from the first day after surgery. This allows for earlier mobilization and protected healing of the PCL, reducing the risk of postoperative knee stiffness. As such, this technique offers advantages over other arthroscopic PCL fixation methods. It allows for optimal visualization, achieves stable fixation, and enables early functional recovery (Table 3).

**Table 3:** Advantages and Limitations

Advantages:
The arthroscopic technique offers several advantages, including a less invasive procedure and the treatment of concomitant meniscal or chondral lesions.
The use of trans-septal visualization enables improved visualization of anatomic landmarks and avoids the need for a 70° arthroscopic camera.
Optimal reduction and stable fixation can be achieved through a combined whipstitching technique using a high-strength ribbon suture in the PCL substance and a high-strength suture tape that embraces the PCL from anterior to posterior and is configured in a crosstie fashion.
Additional fixation protection is provided by InternalBrace ligament augmentation, which allows early recovery of knee function.
Limitations:
Development of the trans-septal portal requires a relatively long learning curve when working with the camera and instruments in a back-and-forth manner.
Fixation is difficult if surgery is not performed within 21 days of injury owing to the development of scar tissue, which can complicate access to the posterior compartment.

PCL, posterior cruciate ligament.

However, it is important to note that TS portal development requires the camera and instruments to work in a back-and-forth manner during the procedure. In cases of PCL avulsion fracture, surgery can be further complicated by the altered anatomy resulting from the injury. Therefore, this technique requires a relatively long learning curve and experienced arthroscopic surgeons.

## Disclosures

All authors (M.C., D.D., L.D., L.B., D.V., F.G., A.M.) declare that they have no known competing financial interests or personal relationships that could have appeared to influence the work reported in this paper.

## Supplementary Data

Video 1 Left knee. Arthroscopic all-inside posterior cruciate ligament (PCL) avulsion fracture suture fixation with double-tunnel pullout and InternalBrace augmentation using trans-septal approach. Preoperative radiographs and computed tomography (CT) and magnetic resonance imaging (MRI) scans are shown. A standard diagnostic arthroscopy is performed. The posteromedial (PM) portal is created via trans-notch visualization. The posterolateral (PL) portal is created in a similar fashion. With the knee constantly maintained at 90° of flexion, an arthroscopic trocar is introduced into the PL portal and advanced toward the posterior septum under direct arthroscopic visualization. The camera is switched to the PM portal, and the trans-septal portal is developed, working with the shaver in a back-and-forth manner from the PL portal. The shaver is directed onto the posterior margin of the tibia and used as a blunt dissector to detach the posterior septal fibers and identify the remaining fibers to be removed. Dissection is carried out slightly distal to the PCL footprint to ensure correct placement of the transtibial tunnels. The PCL footprint is identified and prepared for the fragment’s reduction and fixation. The camera is switched to the PL portal, and a PCL guide handle is used to drill two 3-mm transtibial tunnels at the medial and lateral margins of the PCL footprint with the aid of a cannulated reamer. Two suture shuttles are placed in the tunnels with the aid of a nitinol loop passer. A 45° angled suture hook is used to obtain an intrasubstance PCL stitch with a 1.5-mm high-strength suture ribbon and the aid of a suture shuttle. The tips of the ribbon are retrieved from the PL and PM portals, respectively. The camera is switched to the infrapatellar lateral (IPL) portal. The tip of a high-strength suture tape is passed in the posterior compartment of the knee, in an inter-cruciate manner, and retrieved from the PM portal. The second tip of the suture tape is passed in the posterior compartment of the knee, passing on the medial side of the PCL, thus embracing the ligament remnant. The second tip of the suture tape is retrieved from the PL portal, producing a crosstie configuration of the suture tape on the PCL. With visualization from the IPL portal, a tunnel is drilled at the center of the femoral PCL footprint with the aid of a femoral PCL guide handle and a 4-mm cannulated reamer. A suture shuttle is retrieved from the PL portal in the anterior compartment of the knee. A high-strength suture tape is loaded onto a loop-adjustable cortical fixation device, configuring the InternalBrace ligament augmentation (IBLA). The button is flipped on the medial femoral cortex under direct arthroscopic visualization. The previously positioned suture shuttle is used to retrieve the InternalBrace in the PL portal. The sutures in the PL portal and the IBLA are now retrieved in the lateral tibial tunnel. The same procedure is performed by retrieving the PM portal sutures in the medial transtibial tunnel. The final configuration of the fracture fixation is characterized by the transverse ribbon suture reducing the fracture, the crosstie suture tape compressing the avulsed fragment, and the IBLA system protecting the suture fixation. Optimal reduction and stable fixation can be seen. With the knee maintained at 90° of flexion, a cortical button is used to secure the sutures on the anterior tibial cortex with sliding knots. Final postoperative radiographs are shown.
